# First *LIPA* Mutational Analysis in Egyptian Patients Reveals One Novel Variant: Wolman Disease

**DOI:** 10.1007/s12031-023-02139-6

**Published:** 2023-07-20

**Authors:** Nesma M. Elaraby, Eman Reda Galal, Mohamed Abdel-Hamid, Hasnaa M. Elbendary, Mohamed Elbadry, Mona K. Mekkawy, Neveen A. Ashaat, Samir M. Mounir, Engy A. Ashaat

**Affiliations:** 1 Medical Molecular Genetics Department, Human Genetics and Genome Research Institute, National Research Centre, Cairo, Egypt; 2 Biochemistry and Molecular Biology Department, Faculty of Pharmacy (Girls), Al-Azhar University, Cairo, Egypt; 3 Clinical Genetics Department, Human Genetics and Genome Research Institute, National Research Centre, Cairo, Egypt; 4 Associate Professor of Endemic Medicine Department, Faculty of Medicine, Helwan University, Cairo, Egypt; 5 Human Cytogenetics Department, Human Genetics and Genome Research Institute, National Research Centre, Cairo, Egypt; 6 Professor of Genetics and Biotechnology, Ain Shams University, Cairo, Egypt; 7 Pediatrics Department, Minia University, Minia, Egypt

**Keywords:** Wolman disease, Lysosomal acid lipase deficiency, *LIPA* gene, Novel variant

## Abstract

Lysosomal acid lipase (LAL) is a necessary enzyme for the hydrolysis of both triglycerides (TGs) and cholesteryl esters (CEs) in the lysosome. Deficiency of this enzyme encoded by the lipase A (*LIPA*) gene leads to LAL deficiency (LAL-D). A severe disease subtype of LAL-D is known as Wolman disease (WD), present with diarrhea, hepatosplenomegaly, and adrenal calcification. Untreated patients do not survive more than a year. The aim of this study was to assess the clinical and molecular characterizations of WD patients in Egypt. A total of seven patients (from five unrelated Egyptian families) were screened by targeted next-generation sequencing (NGS), and the co-segregation of causative variants was analyzed using Sanger sequencing. Furthermore, multiple in silico analyses were performed to assess the pathogenicity of the candidate variants. Overall, we identified three diseases causing variants harbored in the *LIPA* gene. One of these variants is a novel missense variant (NM_000235.4: c.1122 T > G; p. His374Gln), which was classified as a likely pathogenic variant. All variants were predicted to be disease causing using in silico analyses. Our findings expand the spectrum of variants involved in WD which may help to investigate phenotype-genotype correlation and assist genetic counseling. To the best of our knowledge, this is the first clinico-genetic study carried out on Egyptian patients affected with WD.

## Introduction

Lysosomal storage disorder is an inherited defect caused by the deficiency of lysosomal acid lipase (LAL) (NP_000226) and is characterized by an accumulation of cholesteryl esters (CEs) and triglycerides (TGs) in the lysosome (Maciejko 2017; Gomaraschi et al. 2018; Li and Zhang 2019; Pastores and Hughes 2020). Deficiency of LAL activity leads to two clinically distinct phenotypes: Wolman disease (WD, OMIM ID: 278,000) and cholesteryl ester storage disease (CESD; OMIM ID: 278,000). WD is a severe and rare fatal metabolic disorder with an incidence rate of less than 1 per 100,000 births (Del Angel et al. 2019). It is presenting through various symptoms such as hepatomegaly, vomiting, splenomegaly, diarrhea, adrenal calcification, failure to thrive, dyslipidemia, and hepatic failure (Abramov et al. 1956). WD usually manifests in the first 2 or 3 months of life resulting from the rapid accumulation of CEs and TGs in tissues. Without treatment, the WD patients die from multi-organ failure within the first year of life (Jones et al. 2016, 2017; Pericleous et al. 2017), while CESD is a mild disorder presenting with elevated liver enzymes, hepatomegaly, and dyslipidemia during childhood or adulthood (Bernstein 2013) with a higher prevalence 1 per 160,000 (Carter et al. 2019). Furthermore, abnormal immune regulation may clarify the features of some metabolic diseases. Hyper-inflammation and pancytopenia, together with hypercytokinemia and hemophagocytic lymphohistiocytosis resulting from immune cell dysfunction, may be observed in WD (Alabbas et al. 2021; Potter et al. 2021; Jordan et al. 2019). Genetic and biochemical evidence indicates that WD and CESD are distinguished by residual lysosomal acid lipase activity.

WD is a rare, autosomal recessive, and fatal disease caused by a significant reduction of LAL activity due to biallelic variations in the *LIPA* gene which is localized on chromosome 10q23.2q23.3, including 10 exons, 9 introns, and encodes (372 amino acid polypeptide) which necessary for secretion and lysosomal targeting of LAL enzyme (Maciejko 2017; Grabowski et al. 2021). As previously mentioned, WD patients possess > 1% of normal LAL levels and CESD is identified by 1–12% of normal LAL activity (Bernstein et al. 2013). These differences in levels of functional LAL depended on the types of mutations at the *LIPA* locus. To date, 130 variants in the *LIPA* gene have been reported in WD and CESD (Strebinger et al. 2019; Parham and Underberg 2021). Of note, more than 12 mutations in the *LIPA* gene have been previously reported for WD. LAL enzyme plays a key role in the hydrolysis of certain fats especially the CEs and TGs into free cholesterol and free fatty acids (Cansever et al. 2019). A deficiency of this enzyme leads to intracellular lipid accumulation especially in the liver, lymph nodes intestine, spleen, and bone marrow (Reiner et al. 2014; Li and Zhang 2019). The definitive diagnosis of WD is done by measuring the level of LAL enzyme activity in fibroblasts or leukocytes and by molecular genetic analysis for *LIPA* gene (Hamilton et al. 2012; Hoffman et al. 2016).

In the present study, we report the clinical, biochemical, and molecular characterizations of seven Egyptian patients with WD referred to our clinic for molecular diagnosis. To obtain a definite genetic diagnosis in all patients, NGS was performed with an extended panel of 41 metabolic related genes including *LIPA* gene. To the best of our knowledge, this is the first clinico-genetic study in WD carried out on Egyptian patients.

## Material and Methods

### Patient Recruitment and Ethical Approval

In this study, seven Egyptian patients descending from five unrelated families clinically and biochemically diagnosed with WD were referred to Clinical Genetics Department, National Research Centre, Cairo, Egypt, for genetic testing. Clinical diagnosis was investigated by a clinical geneticist based on the patient’s laboratory tests and possible phenotypic symptoms. Family history, dysmorphic features, physical examination, and biochemical investigations were evaluated. Pedigrees of families with at least three generations were also performed where possible.

Written informed consent was obtained using a form approved by the Ethics Committee of National Research Centre and was conducted in accordance with the Declaration of Helsinki. Following ethical guidelines, peripheral blood samples were obtained from the patients and their parents for molecular studies.

## Cytogenetic Studies

Karyotyping of all patients was performed from peripheral blood lymphocytes, using Giemsa-Trypsin-G (GTG)-banding technique. Karyotype description followed the International System for Human Cytogenetic Nomenclature recommendations (ISCN 2020).

## Molecular Analysis

### Genomic DNA Extraction

Genomic DNA was extracted from peripheral blood samples of all patients and their available family members using the QIAamp DNA Blood Mini Kit (Qiagen, Hilden, Germany) according to the manufacturer’s instructions. DNA concentration was quantified using a Qubit Fluorometer (Thermo Fischer Scientific Inc.).

## Library Preparation, Sequencing, and Data Analysis

A total of 70 ng/μl DNA of each affected patient was used for sequencing. Design Studio (Illumina) was carried out for library design. A targeted sequencing panel including 41 metabolic genes was performed according to the manufacturer’s instructions (Illumina San Diego, CA, USA). The promoter region and the flanking intronic sequences of the *LIPA* gene (NM_000235.4) are included in the targeted metabolic panel. Sequencing was carried out on MiSeq instruments (Illumina®) using 2 × 150 bp paired-end sequencing. The NGS sequencing protocol was the same as previously reported (Louillet et al. 2018;  Tebani et al. 2021).

Raw image files were processed using FASTQ for base calling and generating raw data. The reads were aligned to the NCBI on human genome (hg19/GRCh37) using the Burrows–Wheeler Aligner tool (BWA-MEM). Variant calling was performed using platform GATK Haplotype Caller. Variants were visualized using integrated genome viewer (IGV). We excluded variants from the variants list that were identified with a frequency of > 1% in the 1,000 Genomes Project (http://browser.1000genomes.org). The filtered variants were compared to variant databases including dbSNP (https://www.ncbi.nlm.nih.gov/snp/), HGMD (http://www.hgmd.cf.ac.uk/), gnomAD (https://gnomad.broadinstitute.org/), and LOVD (https://databases.lovd.nl/shared/). The variants that passed filtering steps were directly confirmed utilizing Sanger sequencing technique, as mentioned below.

## Sanger Sequencing for Variants’ Validation

To confirm the detected variants in the patients and their parents, DNA fragments for *LIPA*-mutated exons were amplified by polymerase chain reaction (PCR) and analyzed using standard Sanger sequencing. The Primer3 tool (http://bioinfo.ut.ee/primer3-/) (accessed on 15 October 2022) was used to design the specific primers for PCR amplification and direct sequencing of the selected candidate causative variants. PCR primer sequences and cycling conditions are available upon request. Amplified fragments were sequenced using ABI 3500 Genetic Analyzer by using the Big Dye Termination kit (Applied Biosystems) according to the manufacturers’ protocols. The sequence files (chromatograms) were aligned and compared with *LIPA* (NM_000235.4) reference sequence by BLAST online software.

## Bioinformatics Prediction Analysis

### In Silico Functional Prediction Tools

Functional predictions were established from sequence homology based on different in silico programs, namely, Mutation Taster (http://www.mutationtaster.org), SIFT (Sorting Intolerant From Tolerant) (http://sift.jcvi.org), and PolyPhen-2 (Polymorphism Phenotyping v2) (http://genetics.bwh.harvard.edu/pph2). Both of the SIFT and PolyPhen-2 tools report results in terms of pathogenic scores as benign, tolerated, or deleterious, whereas Mutation Taster scores classified the detected variants to be disease causing or neutral.

The identified variants were classified into pathogenic, likely pathogenic, uncertain significance, likely benign, and benign according to the American College of Medical Genetics and Genomics and the Association for Molecular Pathology (ACMG/AMP) (Richards et al. 2015).

## Protein Modeling

The target sequences of human LIPA protein were obtained from the UniProt database (https://www.uniprot.org) for homology modeling. FASTA-formatted *LIPA* protein sequences were modeled using SWISS-MODEL (https://swissmodel.expasy.org/) based on the location within the crystal structure of human *LAL* monomer. Finally, protein model with the top score was selected and visualized using PyMOL software (https://pymol.org/).

## *LIPA* Protein Stability Prediction

Protein stability for the mutant *LAL* protein was evaluated using the online tools; I-Mutant v3.0 (http://gpcr2.biocomp.unibo.it/cgi/predictors/I-Mutant3.0/I-Mutant3.0.cgi), Missense3D (http://missense3d.bc.ic.ac.uk/), MuPro (http://mupro.proteomics.ics.uci.edu/), Dynamut (http://biosig.unimelbedu.au/dynamut/), and mCSM (http://structure.bioc.cam.ac.uk/mcsm) are vector machine-based tools that can predict whether an amino acid alteration destabilizes or stabilizes the protein based on the protein sequence. The free energy change (DDG or ΔΔG) value was calculated based on the difference between unfolding Gibbs free energy change of wild-type (WT) and mutant protein (kcal/mol), where the predictions were identified as (ΔΔG value <  − 0.5 kcal/mol) corresponds to decreased stability and (ΔΔG value > 0.5 kcal/mol) means increased stability.

## Analysis of Conserved Residues

ConSurf server (https://consurf.tau.ac.il/) was applied to provide an evolutionary conservation profile for LIPA protein, to better predict the potential disrupting impact of the detected variants. It first detects conserved positions using multiple sequence alignment, then calculates the evolutionary conservation rate by Bayesian inference, and provides the evolutionary conservation profiles of structure or the sequence of the protein. The evolutionary conservation of residues is critical to understand the structure and function of a protein. The ConSurf score represents from 1 to 9, where 1 indicates the least conserved residue, 5 indicates region which is moderate, and 9 is for highly conserved residues.

## Results

### Clinical Findings

**Fig. 1:**
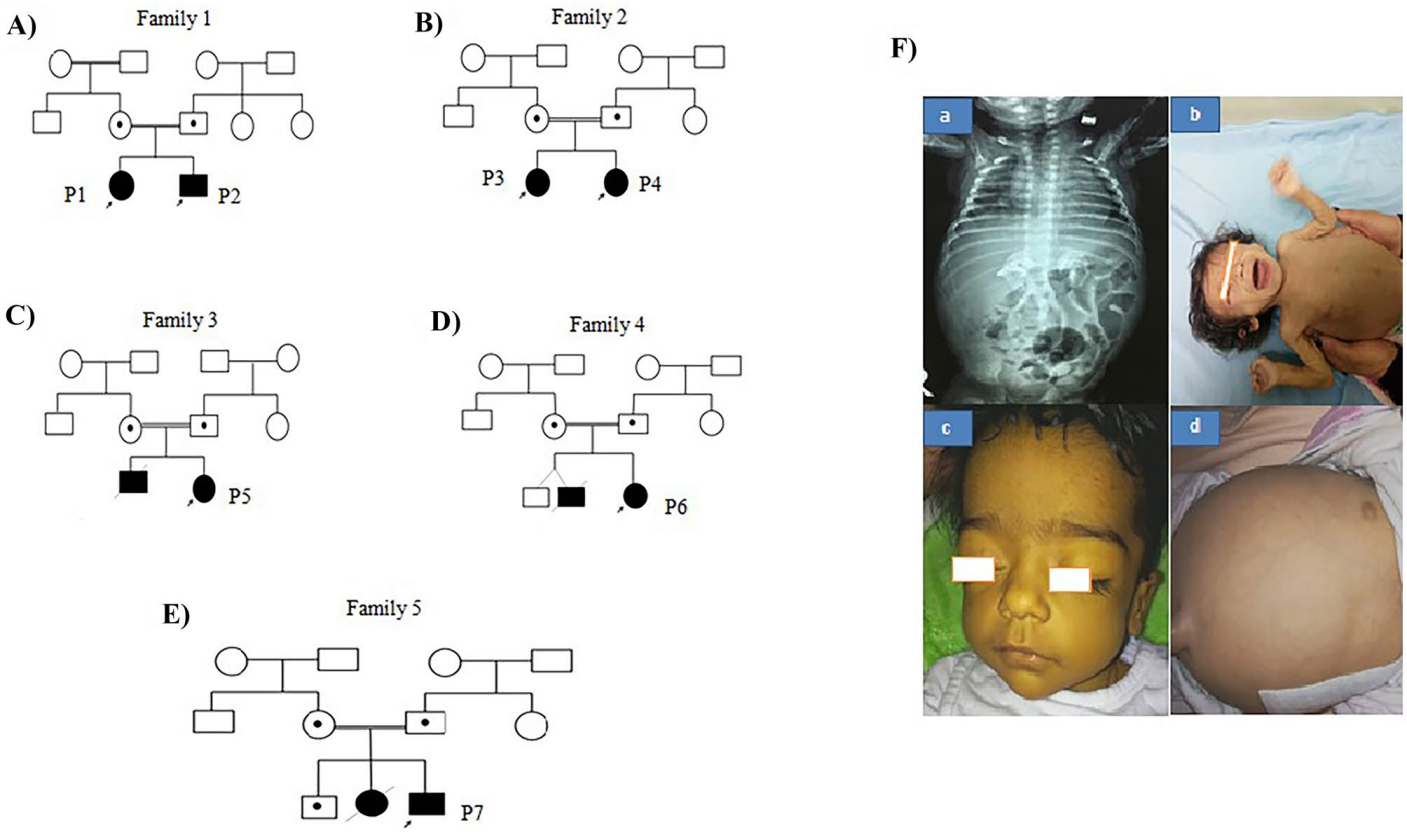
**A–E** Pedigrees of the five families affected with WD. The probands is indicated by a black arrow and given numbers (P1: P7). **F** Clinical phenotype of WD in the five families. (a) Patient 2 at age of 4 months showing coarse facies and lower eyelid puffiness. (a) X-ray chest and abdomen showing bilateral adrenal glands calcification. (b) Patient 3 at age of 3 months showing senile facies and poor weight gain. (c, d) Patient 4 at age of 2 months showing face jaundice, abdominal distention, and umbilical hernia

A total of seven patients (2 males and 5 females) descending from five unrelated Egyptian families with a clinical diagnosis of WD were included in this study. The pedigree provided evidence of autosomal recessive pattern of inheritance. The family history was remarkable for all patients. However, all patients were born to consanguineous parents. History of neonatal similarly affected sibs who died in the 1st year of life. The affected children were diagnosed at early infancy with age varying from 2 to 6 months, which presented with severe abdominal destination, anemia, and low weight gain. Hepatosplenomegaly was also present. All patients showed common gastrointestinal symptoms (GIT) including severe vomiting, steatorrhea, and failure to thrive with the disease progression. Adrenal glands calcifications were confirmed via abdominal radiographs in all patients except P3. Our patients died at the age ranged from 3 to 10 months due to a complicated chest infection and chronic diarrhea. Furthermore, biochemical tests revealed low levels of HDL-C and elevated in LDL-C associated with an increase in plasma TG and TC levels, as well the analysis show increasing in the levels of serum alanine/aspartate transaminases (ALT/AST). The clinical and biochemical characteristics of the affected individuals are summarized in Table 1 and Fig. 1.

**Table 1:** The clinical and biochemical data of WD patients

**-**	**Family 1**		**Family 2**		**Family 3**	**Family 4**	**Family 5**
**Patient ID**	**Patient 1**	**Patient 2**	**Patient 3**	**Patient 4**	**Patient 5**	**Patient 6**	**Patient 7**
**Sex**	M	F	F	F	F	F	M
**Consanguinity**	+	+	+	+	+	+	+
**Family history**	+	+	+	+	+	+	+
**Age at last examination**	4 months	2 months and half	2 months	6 months	3 months	2 months	4 months
**Weight (kg)/SD**	4.5/ − 3.53SD	5.9/ − 0.5SD	3/ − 2.9SD	6/ − 1.75SD	3.5/ − 3.5SD	3/ − 2.5SD	4/ − 4.1SD
**Head circumference (cm)/SD**	40	38.5	39/0.2SD	40.2/ − 1.6SD	36/ − 2.4SD	35/ − 0.3SD	40.5/ − 0.3SD
	− 1.46SD	− 0.5SD					
**Height (cm)/SD**	61/ − 0.87	59/ − 0.5SD	58/0.16SD	62/ − 1.35SD	56/ − 1.4SD	55/0.5SD	60/ − 0.9
**Motor development**	No head support	Motor regression	Motor regression	Motor regression	No head support	No head	No head
**Mental development**	Alert	Alert	Alert	Regression	Regression	Support	Support
**Onset of symptoms**	2 months	2 months	1 month	1 month	1 month	1 month	1 month
**Clinical presentation**	GIT	GIT	GIT	GIT	GIT	GIT	GIT
**GIT symptoms**	Severe	Severe	Severe	Severe	Severe	Severe	Severe
**Diarrhea**	+	+	+	+	+	+	+
**Vomiting**	+	+	+	+	+	+	+
**Steatorrhea**	+	+	+	+	+	+	+
**Abdominal distention**	+	+	+	+	+	+	+
**FTT**	+	+	+	+	+	+	+
**Facies**	Coarse	Coarse	Coarse	Coarse	Senile	Coarse	Coarse
**Hepatomegaly**	+	+	+	+	+	+	+
**LCF manifestations**	Ascites	Ascites	Ascites	Ascites	+	+	+
**Splenomegaly**	+	+	+	+	+	+	+
**Hypersplenism (CBC)**	Anemia	Anemia	Anemia	Anemia	Anemia	Anemia	Anemia
	6 mg/dl	5 mg/dl	4 mg/dl	4 mg/dl	4 mg/dl	6 mg/dl	5 mg/dl
**Blood transfusion**	Frequent	Frequent	+	+	+	+	+
**Adrenal calcifications**	+ bilateral	Bilateral	-	+ bilateral	+	+	+
**Hepatic transaminases**	Elevated	Elevated	Elevated	Elevated	+	+	+
**Serum albumin**	N	N	Hypoalbuminemia	Hypoalbuminemia	Hypoalbuminemia	Hypoalbuminemia	Hypoalbuminemia
**Renal functions**	N	N	N	N	N	N	N
**Acid lipase**	NA	Affected	Affected	NA	Affected	Affected	Affected
**Lipid profile, serum cholesterol**	Elevated	Elevated	Elevated	Elevated	Elevated	Elevated	Elevated
**LDL**	Elevated	Elevated	Elevated	Elevated	Elevated	Elevated	Elevated
**HDL**	Low	Low	Low	Low	Low	Low	Low
**Serum triglycerides**	Elevated	Elevated	Elevated	Elevated	Elevated	Elevated	Elevated
**End stage**	4 months	3 months	6 months	10 months	4 months	3 months	5 months
**Cause of death**	Complicated chest infection, chronic diarrhea	Complicated chest infection, chronic diarrhea	Complicated chest infection, chronic diarrhea	Complicated chest infection, chronic diarrhea	Complicated chest infection, chronic diarrhea	Complicated chest infection, chronic diarrhea	Complicated chest infection, chronic diarrhea

*GIT* gastrointestinal manifestations, *HDL* high-density lipoprotein, *FTT* failure to thrive, *LCF* liver cell failure, *LDL* low-density lipoprotein

Based on these clinical and biochemical characterization, we tended to diagnose our cases to be WD. Genetic tests were carried out on all subjects after the clinical diagnosis of WD to disclose the molecular etiology.

### Molecular Findings

**Fig. 2:**
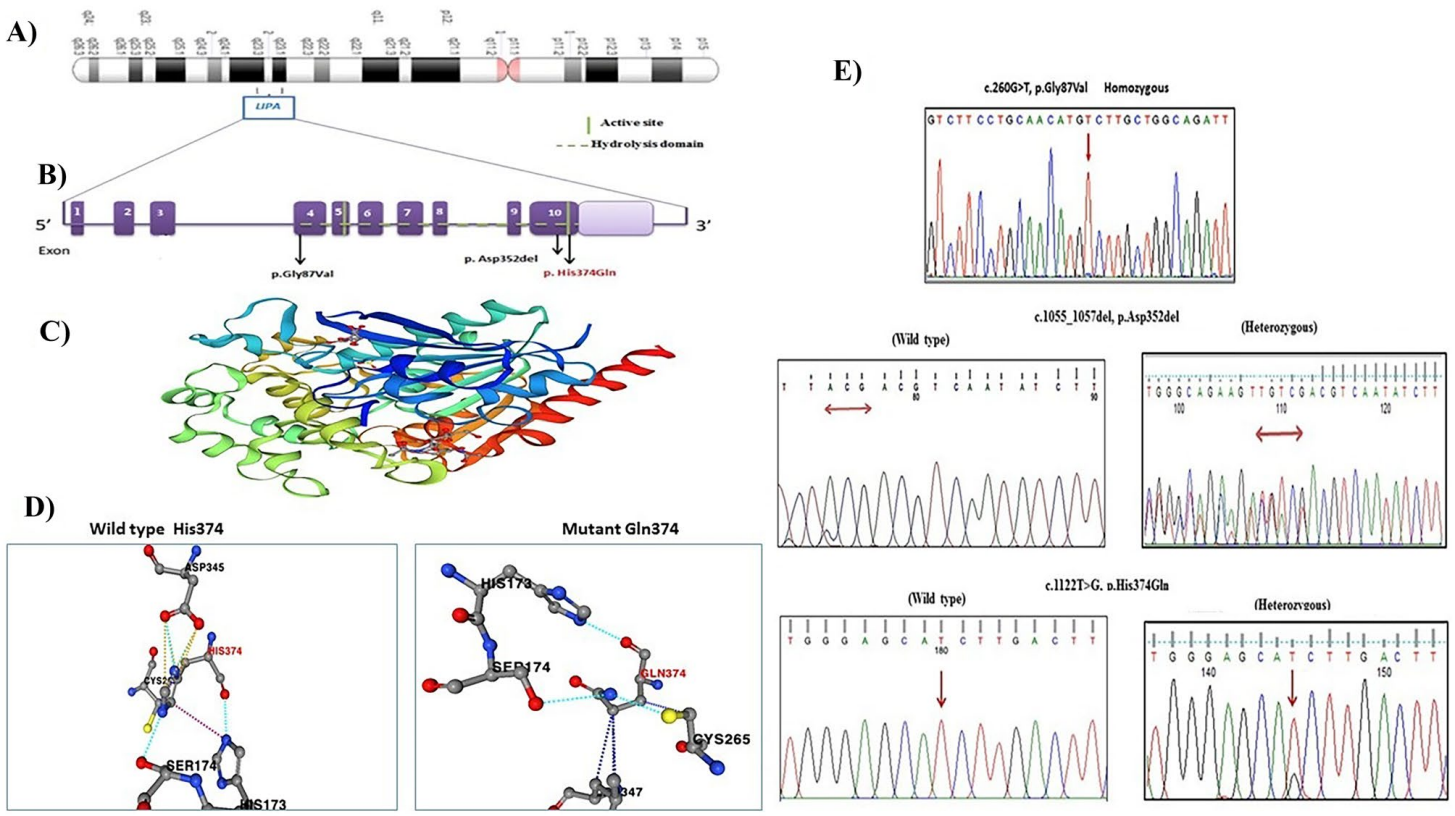
**A** Location of the *LIPA* gene on chromosome 10q23.2q23.3. **B** Diagram of *LIPA* gene showing variants identified in this study. The novel variant is indicated in red. **C** The 3D structure of the *LIPA* protein was predicted by SWISS-MODEL. **D** Wild and mutant residue for the novel missense variant (p.His374Gln) using Dynamut tool. **E** Sanger sequencing chromatogram for the detected variants in available patients (homozygous) or patients’ parents (heterozygous), who completed the follow-up

After filtering the raw NGS data, we were able to identify three homozygous pathogenic variants in the *LIPA* gene sequence (Table 2 and Fig. 2A, E); among them, one is a novel variant c.1122 T > G (p. His374Gln) and two were previously reported (c.260G > T; p.Gly87Val and c.1055_1057del; p.Asp352del). Sanger sequencing validated the three identified variants (Fig. 2E).

**Table 2:** Variants of *LIPA* gene based on online databases and pathogenic prediction tools

**Patient ID**	**Nucleotide alteration**	**Protein alteration**	**Domain location**	**Zygosity**	**Functional prediction tools**				**dbSNP ID**
					**SIFT**	**Polyphen2**	**Mutation Taster**	**Clinical significance ACMG**	
**P1-P2** **P3-P4**	c.260G > T	p.Gly87Val	Exon 4	Homo	Deleterious (0)	Probably damaging (0.999)	Disease causing	Pathogenic (PP5, PP3, PM1, PM2)	rs587778878
**P5- P6**	c.1055_1057del	p. Asp352del	Exon 10	Homo	NA	NA	Disease causing	Uncertain Significance (PM1, PM4, PM2)	rs767207643
**P7**	c1122T > G	p. His374Gln	Exon 10	Homo	Deleterious (0)	Probably damaging (0.995)	Disease causing	Likely pathogenic (PP3, PM1, PM5, PM2)	This study

*P* patient, *Homo *homozygous, *NA* not applicable, *SIFT* Sorting Intolerant From Tolerant, *Polyphen2* Polymorphism Phenotyping v2, *ACMG* American College of Medical Genetics and Genomics

The first *LIPA* disease-causing variant is a well-known missense variant c.260G > T (p. Gly87Val) in exon 4. This variant was reported in two families (1and 2) having four affected members (P1, P2, P3, and P4). The segregation study revealed that c.260G > T variant was inherited from both the normal heterozygous parents.

Notably, the remaining two variants were identified in exon 10 of the *LIPA* gene. One of these variants was already reported c.1055_1057del (p. Asp352del). This variant was identified in two probands (P5, P6) from family 3 and family 4, respectively. Sanger sequencing of this variant Asp352del was performed in their parents to confirm the presence and pattern of inheritance. As well, this variant was reported in gnomAD database only in heterozygous state, while the second one is a novel missense variant c.1122 T > G (p. His374Gln), which was identified in family 5 affected member (p7). This variant has not been reported in the previous studies or presented in the databases (1,000 genome project, genome AD, and clinvar) or in our in-house databases of 55 Egyptian exomes.

### Bioinformatics Prediction

As we mentioned in Table 2 and according to our in silico analysis, all detected missense variants in the transcript (ENST00000336233.10) of *LIPA* gene were evaluated for its pathogenicity and were considered to be disease-causing variants, deleterious, and probably damaging using Mutation Taster, SIFT, and Polyphen-2 programs, respectively. Thus, none of these detected variants was expected to be benign.

According to the ACMG guidelines for variant interpretation, the two missense variants (c. 1122 T > G and c.260G > T) could be classified as likely pathogenic and pathogenic, respectively, while the c.1055_1057del variant was classified to be variant of uncertain significance (VUS) with unknown effect based on the evidence chain (PM1, PM4, PM2), respectively (Table 2).

The human LIPA amino acid sequence was obtained in FASTA format from UniProt (ID P38571). The 3D structure of the LIPA protein was modeled based on the X-ray structure with PDB code: 6v7n.1.A which was the best template for the model and has a sequence identity of 98.94% with the LIPA protein. The 3D structure of the protein was predicted by SWISS-MODEL method as given in Fig. 2C.

The impact of the two missense variants on protein stability changes was studied to evaluate its impact on LIPA protein folding. The change of unfolding Gibbs free energy (DDG or ΔΔG) was calculated using different online tools as described in (Table 3). Based on the results obtained from these algorithms, it was shown that the two missense variants (p. Gly87Val and p. His374Gln) had a damaging effect on the LIPA protein structure stability (decreasing its protein stability). Moreover, the p.Gly87Val variant demonstrated a mild damaging effect on LIPA protein stability, as its ΔΔG values were closer to zero (− 0.52, 0.006, − 0.496, and 0.195 kcal mol^−1^), respectively, in I-Mutant 3.0, MUPro, mCSM, and Dynamut tools. Regarding p.His374Gln, this substitution replaces a buried charged residue with an uncharged residue (Gln) and disrupts a salt bridge formed by ND1 atom of His 374 and OD2 atom of Asp345 (distance: 2.606 Å). The wild-type residue has RSA of 0.0% (His, RSA 0.0%) (Fig. 2D).

**Table 3:** Change of unfolding Gibbs free energy due to the detected missense variants

Variant	I-Mutant		MUPro		mCSM		Dynamut	
	**Stability**	**DDG**	**Stability**	**DDG**	**Effect**	**ΔΔG**	**Effect**	**ΔΔG**
p.Gly87Val	Decrease	− 0.52 kcal mol^−1^	Increase	0.006 kcal mol^−1^	Destabilizing	− 0.496 kcal mol^−1^	Stabilizing	0.195 kcal mol^−1^
p. His374Gln	Decrease	− 0.32 kcal mol^−1^	Decrease	− 0.74 kcal mol^−1^	Destabilizing	− 2.195 kcal mol^−1^	Destabilizing	− 0.363 kcal mol^−1^

ΔΔG/DDG Gibbs free energy change score indicated increases in protein stability > 0 > decrease in protein stability

**Fig. 3:**
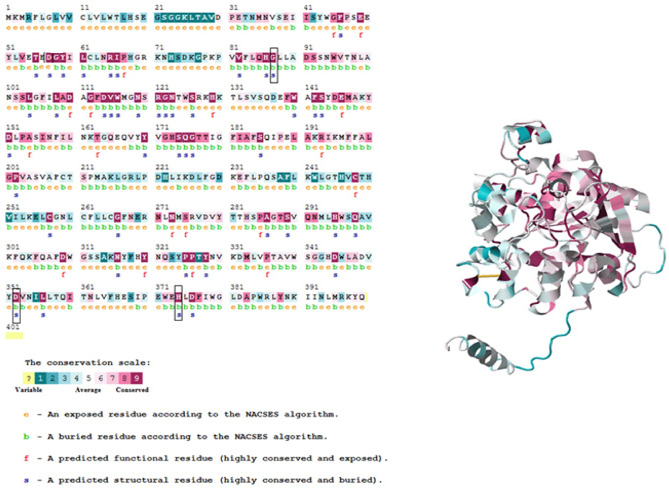
Conservation analysis of the LIPA protein using ConSurf web server. The ConSurf analysis is implemented for 399 residues of LIPA protein and indicated by definite score represents from 1 to 9. The small rectangular boxes show the mutant residues detected in this study

Analysis of amino acid residue conservation in LIPA protein structure is used to understand its importance and localized evolution. Mutations in the conserved region of the protein (ConSurf score from 7 to 9) are expected to be more damaging compared to those in the less conserved region. As shown in Fig. 3, all the detected variants in this study were found in the highly conserved regions with conservation scores ranging from 7 to 9. As well, three residues (p.Gly87Val, p. Asp352del, and p. His374Gln) were predicted to be buried and structural; the buried residues with high scores usually play a role in maintaining the structural integrity while the exposed residues are considered to be functional residues.

## Discussion

As we mentioned previously, WD is a lysosomal storage autosomal recessive disorder caused by LAL-D, due to a mutation in the LIPA gene. After molecular analysis, NGS allowed the identification of the molecular basis for WD in seven cases which confirmed the presence of three different homozygous disease-causing variants in the LIPA gene expected to be deleterious for the LIPA protein using different in silico prediction tools. Among these, one is novel and two were previously reported. We confirmed the segregation of these variants with Sanger sequencing. The affected patients were diagnosed between 2 and 6 months of life presenting with severe clinical signs including abdominal distension, hepatomegaly, splenomegaly, gastrointestinal disturbances, anemia, adrenal calcification, failure to thrive, and premature death. Regarding biomedical testes, we investigated that abnormal serum lipid profiles display high levels of serum TC, TG, and LDL-cholesterol in all cases (hypercholesterolemia) and elevated serum transaminase levels ALT/AST.

In this study, to identify the underlying genetic variants associated with WD, we performed a targeted NGS metabolic panel. One of the most important aspects of NGS panel is its ability to easily find the causal variants or even novel genes associated with human diseases. Moreover, compared with whole exome sequencing (WES), analysis of the targeted multigene panel is easier and faster.

Previous studies have attempted to compare the clinical characteristics of patients with WD. Our patients and those described by Ruiz-Andrés et al. and Mandadzhieva et al. (Ruiz-Andrés et al. 2017; Mandadzhieva et al. 2017) seem to have the same clinical disease. Regarding biochemical results, it is consistent with the previous literatures which reported that WD patients revealed hypercholesterolemia and high transaminase levels (Shome et al. 2002; Low et al. 2004; Bernstein et al. 2013; Shenoy et al. 2014).

In line with the importance of LAL enzyme, both Reiner et al. and Koranantakul et al. have reported that the lack of this enzyme leads to the accumulation of both CEs and TGs in different body organs causing several cellular damages such as in the liver (microvacuolar steatosis, increased transaminase levels, fibrosis, and cirrhosis), cardiovascular system, and spleen (splenomegaly), and gastrointestinal disturbances including diarrhea and abdominal pain are related to lipid accumulation in the intestinal mucosa (Reiner et al. 2014; Koranantakul et al. 2021; Patrick and Lake 1969).

Similarly, LIPA is a highly conserved gene, and its hydrolase domain is similar to other lipases. Most LIPA gene variant results due to reduce or loss in LAL activity in WD patients, the majority of these variants (42%) are deletions/insertions and the rest are missense and splice-site variants (Bernstein et al. 2013). In this study, the genetic analysis revealed a homozygous substitution in exon 4 in the LIPA gene, which found in four patients descending from two families, namely, c.260G > T. These variants substitute glycine at position 87 with valine amino acid, in which valine side chain interferes with the active-site serine residue and partly blocks the access to the presumed TG binding site of the lipase. Thus, this substitution leads to loss the enzymatic activity of the lipase (Mandadzhieva et al. 2017; Zschenker et al. 2001) The c.260G > T has been previously reported both in probands with WD and in those with CESD and was classified as pathogenic (Pagani et al. 1996, 1998; Valles-Ayoub et al. 2011; Consuelo-Sánchez et al. 2019). Interestingly, Valles-Ayoub et al. detected the p. Gly87Val in Iranian-Jewish (IJ) children and reported that it may be a founder variant responsible for most WD IJ cases (Valles-Ayoub et al. 2011).

Notably, two variants were reported in exon 10 of LIPA gene. One of these variants c.1055_1057del, p. (Asp352del), was identified in two patients. The c.1055_1057del variant has been previously described in genome AD database but given a very low frequency in the ExAC with no homozygous individuals for the alternate allele. Ruiz-Andrés et al. also reported CESD patient who carried the same variant in the heterozygous form (Ruiz-Andrés et al. 2017). This variant results in the deletion of an Asp at position 352 in the highly conserved region. The other variant (p.His374Gln) is identified for the first time and was predicted to be a likely pathogenic variant according to the AGMG guidelines. All in silico analyses agreed that the novel substituted (p.His374Gln) in the active site might destabilize the protein structure at a conserved site given a negative score. Thus, this variant might lead to a major disruption in LIPA protein structure, but further studies are needed to reveal the exact molecular mechanisms that contribute to the pathogenicity of this novel variant.

Several studies suggested that in silico prediction tools might be used as a first-line molecular diagnosis tools serving both genetic counseling and variant classification (Richards et al. 2015; Parsamanesh et al. 2019). Prediction of the missense variants’ impact on the stability of LIPA protein structure is a critical aspect for studying the function of the protein. Our findings from these predictors classified the detected variants as deleterious, probably damaging, and disease-causing variants.

The correlation between LAL phenotype and genotype is still a matter of disputation in WD and CESD cases. It is interesting to note that the reported variants of WD (p.Gly87Val and p. Asp352del) have been previously reported in CESD patients (Pagani et al. 1998; Ruiz-Andrés et al. 2017).

## Conclusion

We herein confirm the utility of NSG metabolic panel in expanding genotype–phenotype correlations through the identification of one novel and three previously reported variants in the LIPA gene in Egyptian patients with WD. We hope that this identification may reveal new insights into the mechanisms of Wolman disease, mainly in relation to metabolic alterations occurring during this process. To the best of our knowledge, no similar studies have been performed previously in WD Egyptian patients.

## Data Availability

All data from this research can be made available upon request from the corresponding author.
